# Vibrational spectroscopy identifies myocardial chemical modifications in heart failure with preserved ejection fraction

**DOI:** 10.1186/s12967-023-04465-0

**Published:** 2023-09-11

**Authors:** Leonardo Pioppi, Reza Parvan, Alan Samrend, Gustavo Jose Justo Silva, Marco Paolantoni, Paola Sassi, Alessandro Cataliotti

**Affiliations:** 1 Department of Chemistry, Biology and Biotechnology, University of Perugia, 06123 Perugia, Italy; 2 Institute for Experimental Medical Research, Oslo University Hospital and University of Oslo, Oslo, Norway

**Keywords:** FTIR imaging, Micro-Raman spectroscopy, HFpEF, Heart tissue, Tissue print

## Abstract

**Background:**

Vibrational spectroscopy can be a valuable tool to monitor the markers of cardiovascular diseases. In the present work, we explored the vibrational spectroscopy characteristics of the cardiac tissue in an experimental model of heart failure with preserved ejection fraction (HFpEF). The goal was to detect early cardiac chemical modifications associated with the development of HFpEF.

**Methods:**

We used the Fourier-transform infrared (FTIR) and Raman micro-spectroscopic techniques to provide complementary and objective tools for the histological assessment of heart tissues from an animal model of HFpEF. A new sampling technique was adopted (tissue print on a CaF_2_ disk) to characterize the extracellular matrix.

**Results:**

Several spectroscopic markers (lipids, carbohydrates, and glutamate bands) were recognized in the cardiac ventricles due to the comorbidities associated with the pathology, such as obesity and diabetes. Besides, abnormal collagen cross-linking and a decrease in tryptophan content were observed and related to the stiffening of ventricles and to the inflammatory state which is a favourable condition for HFpEF.

**Conclusions:**

By the analyses of tissues and tissue prints, FTIR and Raman techniques were shown to be highly sensitive and selective in detecting changes in the chemistry of the heart in experimental HFpEF and its related comorbidities. Vibrational spectroscopy is a new approach that can identify novel biomarkers for early detection of HFpEF.

**Supplementary Information:**

The online version contains supplementary material available at 10.1186/s12967-023-04465-0.

## Background

It is widely accepted the idea that physiological and pathological conditions can be detected by probing the optical properties of cells and tissues to assess changes in morphology, mechanics, and biochemistry [1]. Although histopathology and immunohistochemistry are currently the gold standards for determining the pathophysiological status of biological samples, the need for new diagnostic tools arises to overcome their limitations [2]. Histological methods rely exclusively on ex-vivo biopsies, which require time-consuming manipulation steps thus causing delayed results [2]. It is necessary to use many chemicals for fixation, inclusion, and staining procedures; these can induce physicochemical changes that significantly alter the structure and composition of the biological specimen of interest, increasing the risk of contamination [3]. Additionally, staining a tissue section can only be used to monitor a few components at once; a complete evaluation requires serial staining and sectioning steps, resulting in bigger sample sizes and longer diagnostic times.

Optical techniques, and specifically vibrational spectroscopies based on infrared (IR) absorption or Raman scattering, offer an alternative to histopathology with some advantages. Besides being non-invasive, non-destructive, and label-free, the Fourier Transformed InfraRed (FTIR) and Raman spectra rely on the total biochemical composition of a sample, revealing the fingerprints of multiple substances in a single measurement [4]. Vibrational spectra can provide useful information not only about the chemical composition of a biological sample but also about its structural properties. This high sensitivity to monitor biochemical and structural changes likely allows to reveal the onset of pathologies even before the morphological and systemic manifestation of the disease [4, 5].

The application of FTIR and Raman spectroscopy to the diagnosis of cardiovascular diseases has been poorly investigated [6–9]. Recent research from Tombolesi et al. [4] has shown the advantages of the combined use of these techniques in the characterization of cardiac tissue and early diagnosis of heart failure. Heart failure (HF) is a complex clinical syndrome resulting from structural and functional impairment of ventricular blood filling or ejection. There are currently about 64 million people suffering from HF worldwide, and the number continues to rise [10]. HF with preserved ejection fraction (HFpEF) is a medical condition characterized by cardiac remodeling that, at an advanced stage, evolves towards concentric hypertrophy, induces left ventricular (LV) wall thickening and changes in the volume and mass of the LV. Studies show that more than half of all patients with HF are affected by HFpEF [11, 12], which is insidious to recognize due to the plethora of risk factors and comorbidities associated with the pathology. The comorbidities accompanying HFpEF are essentially non-cardiac conditions, such as obesity, diabetes mellitus, hypertension, anaemia, chronic obstructive pulmonary diseases, and chronic kidney diseases. It is a complex scenario, in which it is important to recognize the different chemical alterations, secondary to comorbidities, that lead to structural and functional abnormalities of the heart.

In this work, we used both FTIR and Raman spectroscopic methods to investigate heart tissues from lean and obese ZSF1 rats which are an established model of HFpEF with combined comorbidities. The main objective was to detect cardiac chemical modifications associated with the development of HFpEF. Changes in the average biochemical composition of the tissue from obese compared to lean rats were related to the advanced stage of HFpEF and cardiac remodeling observed by echocardiography. We monitored the effect of obesity and diabetes as comorbidities, in this respect we compared spectroscopic results with biochemical and histochemical data. To assess the properties of the extracellular matrix (ECM), we analyzed the tissue print on a substrate. This procedure was previously used for the identification of ECM in kidney cancer [13] and was never applied to the heart. We employed this approach to verify whether the analysis of ECM, other than the whole tissue, provides spectroscopic markers of HFpEF. Once recognized the spectral markers of the pathology, IR and Raman techniques were used to monitor the early stage of the pathology, thus overcoming the limitations of conventional diagnostic tools.

## Methods

### Study protocol

Eight four-week-old male ZSF1 Obese (Ob) and eight lean (Ln) were purchased from Charles River Laboratories (USA). Rats were housed in a room with a 12/12-h light cycle, temperature of 21 °C, and humidity of 55%. Rats were maintained on regular chow, water and food were provided ad libitum for the entire course of the study. At the age of 25 weeks, when obese rats developed HFpEF, were euthanized according to American Veterinary Medical Association (AVMA) Guidelines for the Euthanasia of Animals (2020) via deep anaesthesia (5% isoflurane), exsanguination and organ excision. Due to obvious phenotype (Ob vs Ln) blindness was not possible for in vivo assessments. However, blinding was performed for all the ex vivo assessments.

### Ultrasounds

Cardiac function was assessed the day before sacrifice by transthoracic echocardiography using a VEVO 3100 high-resolution in vivo imaging system from VisualSonics (Amsterdam, NL). Briefly, animals were maintained under anesthesia (1.5–2% isoflurane mixed with oxygen) on a pre-warmed ECG transducer pad with body temperature and ECG monitored. Measurements were made with an MS250 transducer, frequency set at 20 MHz. B-mode measurements in the parasternal long axis view were obtained to assess the function and dimension of the left ventricle (LV). LVEF was calculated as 100 * ((LV Vol;d—LV Vol;s) / LV Vol;d). E and A waves in LV filling velocities were assessed via pulsed-wave Doppler in the parasternal long axis view.

### Biochemical assessments

Samples were sent in one batch for a one-kit analysis of blood using an enzymatic method on an automatic biochemical analyzer (Hitachi 7080, Tokyo, Japan). Glucose, cholesterol, and triglycerides were measured in serum.

### Blood pressure and hemodynamic measurements

Before sacrifice, animals were placed over a heating platform (preheated to 33 to 35 °C), intubated and kept under anaesthesia with a mix of 2.5% isoflurane and 97.5% oxygen using the VentElite small animal ventilator (Harvard Apparatus). Invasive measurements were done using Powerlab/Millar modules (ADInstruments) and the SPR-838 rat pressure–volume catheter. Via closed chest approach, arterial pressure recordings were assessed at the level of the right carotid artery. All the analyses were performed in LabChartPro (v8.1.19) following the PV-loop workflow. Pressure calibration was performed before each recording and 10 mmHg was then added to the channel to correct for a systematic error.

### Histochemistry

Hearts were excised, rinsed in PBS, quickly blotted on gauze, and then fixed in 10% formalin for 24 h. The biventricular apex of the heart was embedded in paraffin and cut in 4 µm sections. Sections were stained with Masson’s trichrome (Polysciences, Inc., Warrington, PA, USA) to assess collagen abundance. Stained sections were scanned (20 × magnification) with AxioScan Z1 (Carl Zeiss, Jena, Germany), to obtain whole cross-sections for collagen quantification. Total fibrosis area (%) and perivascular fibrosis (ratio of the area of fibrosis surrounding the vessel wall to the lumen area) were quantified using ZEN2 blue edition (Carl Zeiss). All histological quantifications were independently performed by trained researchers blinded to groups.

### Samples for spectroscopic analyses

Snap-frozen sections of murine cardiac ventricles were used. Ventricular sections of a total of six replicas of Ln and Ob tissues were analyzed by Raman and FTIR spectroscopies. In addition, a tissue-print procedure was applied to each sample to further extend the analysis of these non-fixed specimens. In particular, RV and LV sections were impressed on CaF_2_ windows to get the deposition of thin films of the extracellular matrix that were inspected by FTIR in transmission mode. Since the heart tissue is homogeneous on the spatial scale of the resolution achievable with our experiments, mean spectra characteristic of different types of tissues and films (Ln and Ob) were compared.

### Raman measurements

Raman scattering spectra were recorded with an Olympus IX73 inverted confocal microscope coupled to the S&I MonoVista CRS + spectrometer, directly on snap-frozen tissue sections of cardiac ventricles. An excitation wavelength of 785 nm, with a power of 13.6 mW, was used in backscattering mode, along with a grating of 300 groves/mm and a 10 × objective lens (N.A. = 0. 30). Each spectrum was the average of 60 accumulations at 10 s integration time in the 400–1942 cm^−1^ range, with a spectral resolution of ∼2 cm^−1^. Spike removal was performed by Origin 2022 software from OriginLab.

### FTIR-Attenuated Total Reflection (ATR) measurements of non-fixed cardiac tissue

FTIR-ATR spectra were collected directly on the LV and RV of heart tissues by using a Bruker Tensor 27 spectrometer and a Hyperion 3000 microscope equipped with a 20 × ATR-objective, which is provided with a germanium crystal, and a 64 × 64 pixel nitrogen-cooled focal-plane-array detector. The 900–3800 cm^−1^ spectral range was collected with a spectral resolution of 6 cm^−1^ and 512 scans. FTIR images (4096 spectra for each map) were collected in two different regions of each ventricle and corrected with the proper atmospheric compensation routine of OPUS 8.1 from Bruker Optics.

### FTIR measurements of tissue print (ECM film)

ECM films were analyzed in transmission mode by FTIR spectroscopy. Spectra were collected using a Bruker Tensor 27 spectrometer and a Hyperion 3000 microscope equipped with a 15 × Cassegrain objective and a nitrogen-cooled single-element mercury cadmium telluride detector. For every sample, 75 randomly selected punctual measurements were performed and the spectral range from 750 to 7500 cm^−1^, with a spectral resolution of 6 cm^−1^ and 512 scans, was recorded. All the spectra were corrected with the atmospheric compensation routine of OPUS 8.1, and a Standard Normal Variate Normalization in the range 900–1800 cm^−1^ was applied. FTIR spectra were analyzed as second derivative profiles.

### Statistical analysis

Raman spectra were obtained from 20 different points of each tissue sample. All the following spectral manipulations were conducted in R-studio. A quality test was carried out to identify and discard poor quality data: spectra selection was done by setting a minimum threshold equal to 1/10 of the maximum integral intensity of the amide I band. A baseline correction was carried out by means of a fifth-order polynomial method. A frequency calibration was performed, setting the intense phenylalanine peak at 1003 cm^−1^. Spectra were normalized to the intensity of the same 1003 cm^−1^ peak area. To test the reproducibility of data, a Principal Component Analysis (PCA) was applied. A single PC does not necessarily represent a single molecular species, since different types of spectra can be distinguished by the variation of several bands with respect to others. The molecular species constituting the tissue were assigned to these bands and the dominant PC1, PC2 and PC3 axes were considered to identify outliers. The two groups of cardiac tissues from Ln and Ob rats are well discriminated using this method. Once the outliers were discarded from the dataset, at least 60 Raman acquisitions for each type of tissue, RV and LV from Ln and Ob rats, were averaged to obtain the mean Raman spectrum with corresponding standard deviation.

FTIR images were collected in two different regions of each ventricle. These regions were randomly selected over the entire sample area in order to obtain a spectrum that is representative of the whole sample. A quality test was carried out for the FTIR spectra to remove spectra with a low signal-to-noise ratio. The absorbance value of the amide I band (1620–1690 cm^−1^) was considered as the reference signal, while the absorbance values in the signal-free region (1800–1900 cm^−1^) as the noise. Spectra were selected by setting a minimum threshold equal to 1/10 of the maximum integral intensity of the amide I band and a signal-to-noise ratio higher than 80. Data were analyzed as second-derivative profiles. A Standard Normal Variate Normalization in the range 900–1800 cm^−1^ was executed to normalize spectra. As well as with Raman spectra, a mean (second derivative) profile and corresponding standard deviation was obtained for each sample type (LV and RV from both Ln and Ob rats).

The difference profiles obtained from Raman spectra were obtained by subtracting the Ln from the Ob data: in this way, we obtained positive features for those signals with a higher intensity in Ob samples. For IR measurements (both ATR-FTIR of tissues and transmission spectra of ECM films), the difference was evaluated by subtracting the Ob from the Ln spectra. In the second derivative spectra, the negative sign of IR spectral components determined the different choice, i.e. opposite sign in the difference. In this way, if the Ob-Ln difference of Raman spectra is compared to the Ln-Ob difference of IR second derivative profiles, the same interpretation can be adopted: a positive feature is related to an increase of intensity in Ob with respect to the Ln counterpart. For the interpretation of the spectra, we referred to [7, 14–16].

## Results

### ZSF1 rats: functional and structural assessment of the heart

**Fig. 1:**
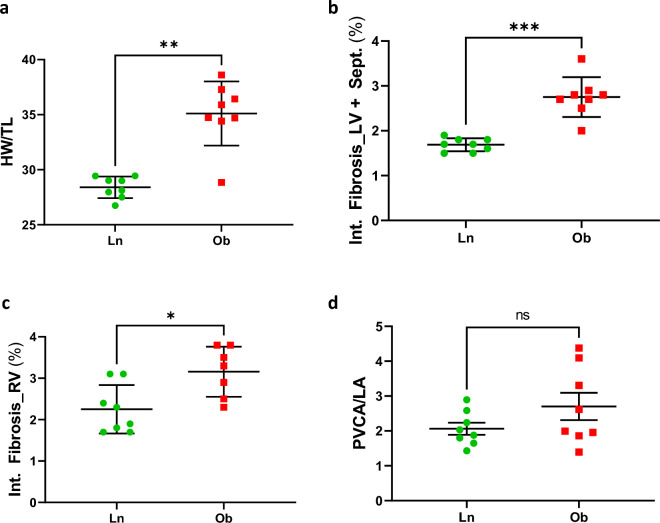
Cardiac structure and histology. (**a**) Heart weight to tail length (HW/TL); interstitial fibrosis (Int. Fibrosis) of (**b**) left (LV) and (**c**) right (RV) ventricles; (**d**) Perivascular collagen area to lumen area (PVCA/LA). Eight lean rats (Ln) and eight obese rats (Ob) were studied. Values are expressed as mean ± SD. * P < 0.05 compared to Ln; ** P < 0.005 compared to Ln; *** P < 0.001 compared to Ln; *ns* = non-significant

As previously described [17], Ob rats exhibited a remarkable increase in body weight, elevated levels of circulating glucose, triglycerides, cholesterol, and lipids, as well as elevated blood pressure compared to Ln rats (Additional file 1: Figure S1A–G). Furthermore, echocardiographic examinations have shown a significant increase in the E/A ratio in Ob rats compared to Ln rats (as shown in Additional file 1: Figure S1H). Meanwhile, the EF of Ob rats remained unchanged (as shown in Additional file 1: Figure S1I). Additionally, our study has demonstrated that the ZSF1 rat model successfully replicated the most crucial clinical features of patients with HFpEF, such as concentric hypertrophy, diastolic dysfunction, and metabolic syndrome/diabetic phenotype (as shown in Fig. 1 and Additional file 1: Figure S1).

In Ob rats, cardiac hypertrophy was evidenced by a 77% increase in heart weight (Fig. 1a). Interstitial heart fibrosis in both LV and right ventricle (RV) was slightly elevated in Ob compared to Ln rats (Fig. 1b, c). Perivascular collagen area to lumen area (PVCA/LA) appeared to be similar between Ln and Ob rats (Fig. 1d).

### Spectroscopy

#### Principal component analysis of Raman data

**Fig. 2:**
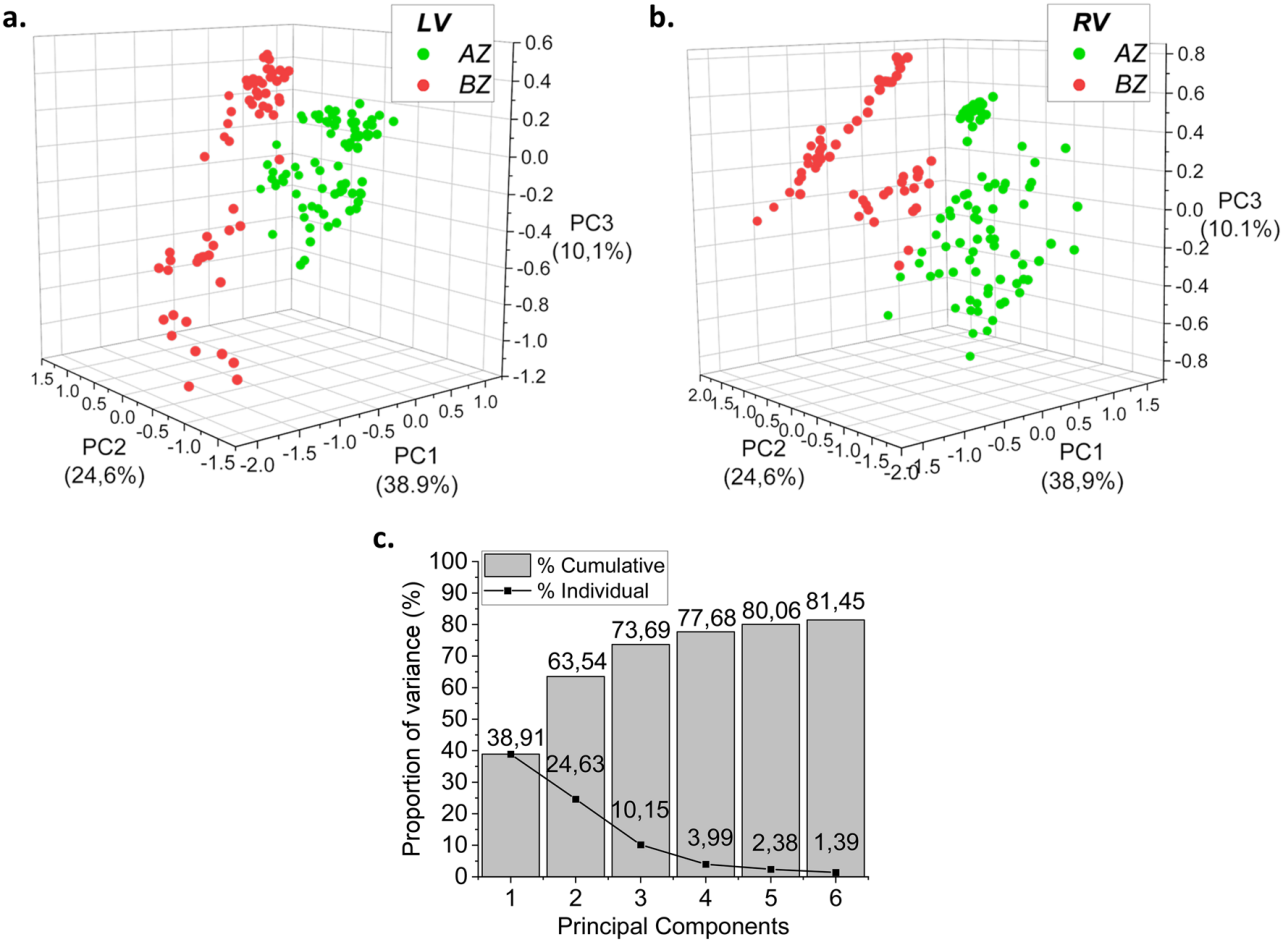
Principal component analysis of Raman data. Scores of PC1, PC2 and PC3 for the left (**a**) and right (**b**) ventricles. Cumulative and individual variances of the first six components of PCA (**c**). Data were collected from heart tissues of six lean rats and six obese rats

RV and LV tissues taken from Ln and Ob animals were studied. A PCA was executed to better identify which spectral features were involved in the differentiation between tissues from Ln and Ob rats (a detailed description of these principal components can be found in the Additional file). The score plots (Fig. 2a, b) showed that, for both RV and LV, the two populations are clearly separated by the three main principal components, which describe more than 73% of the variance (Fig. 2c) in the dataset.

In the case of the LV, the separation is entirely dependent on PC1. Specifically, PC1 loading (Additional file 1: Figure S2A) shows positive contributions at 1238 and 1577 cm^−1^, and negative ones at 850, 932, 1046, 1300, 1451 and cm^−1^. Ln and Ob samples are grouped at positive and negative PC1 scores, respectively. PC2 represents more than 22% of the variance of the spectral dataset and it is characterized by predominantly negative contributions at 1259, 1340, 1441, 1605, and 1655 cm^−1^ (Additional file 1: Figure S2B). Differently from the LV, the PCA analysis on spectra of the RV showed that Ln and Ob keep clustering up to PC3. Specifically, Ob samples have positive scores, whereas Ln scores are almost exclusively negative. PC3 loading shows negative contributions mainly at 1178 cm^−1^, while positive ones are at 841, 915, 971, 1049, 1229, and 1458 cm^−1^ (Additional file 1: Figure S2C). The combination of the spectral contributions of PC1, PC2, and PC3 to the clear separation of Ln from Ob suggests the development of different modifications of the biochemical composition of RV compared to LV.

#### Mean Raman spectra and different profiles of heart tissue

**Fig. 3:**
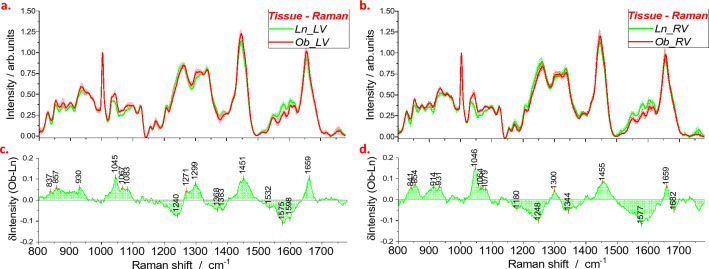
Mean Raman spectra obtained from tissues of the left (**a**) and right (**b**) ventricles of Ln and Ob rats; standard deviations are reproduced by shadowed areas. Difference profiles in panels (**c**) and (**d**) are obtained by subtraction of the green from the red curve of panels (**a**) and (**b**), respectively. Data were collected from heart tissues of six lean rats and six obese rats

Data selected by PCA (a few outliers were eliminated) were averaged to obtain mean spectral profiles representative of LV (Fig. 3a) and RV (Fig. 3b) of Ln and Ob samples. To better highlight variations of spectral profiles, we also evaluated the difference spectrum, obtained by subtracting the mean spectrum of the Ln from one of the Ob samples. The results are shown in Fig. 3c, d. The positive contributions to the difference spectrum identify the wave numbers at which the signal intensity is higher in the Ob samples than in the Ln ones. Negative contributions, on the other hand, are found in bands where the intensity is higher in Ln samples than in Ob ones. Difference profiles show that the most significant variations in signal intensity from spectra of Ln to Ob samples are positive. In particular, we can identify three spectral regions in which positive contributions appear for both LV and RV tissue spectra: 830–950 cm^−1^; 1030–1100 cm^−1^; and 1420–1480 cm^−1^. A positive narrow contribution centered at 1660 cm^−1^ is particularly intense for the different profiles of the LV (Fig. 3c). Negative contributions are observed in the 1150–1260 cm^−1^ and 1500–1625 cm^−1^ spectral ranges (Fig. 3c, d).

#### Mean Attenuated Total Reflection (ATR)-FTIR spectra and different profiles of heart tissues

**Fig. 4:**
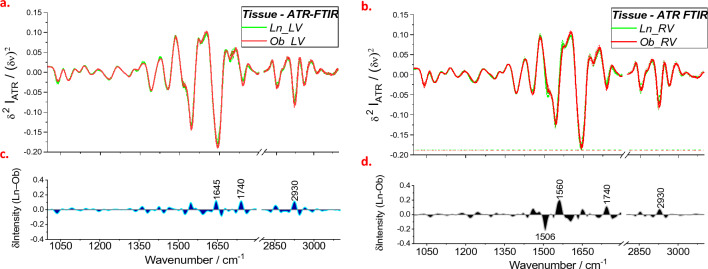
Mean ATR-FTIR spectra obtained from tissues of the left (**a**) and right (**b**) ventricles of Ln and Ob rats. Difference profiles in panels (**c**) and (**d**) are obtained by subtraction of the green from the red curve of panels (**a**) and (**b**), respectively. Data were collected from heart tissues of six lean rats and six obese rats

To further inspect the average composition of Ln and Ob tissues, the same samples investigated by Raman spectroscopy were analyzed by ATR-FTIR. The average ATR spectra of LV and RV samples obtained from Ln and Ob groups are shown in Fig. 4 as second derivative spectra. In this representation, the intensity of a spectral component is more and more negative when increasing the concentration of the molecular species assigned to the band. Figure 4a, b show both data groups with their standard deviations. Intensity variations from Ln to Ob are hardly detected; the few significant changes are larger for the RV and particularly in the 1480–1600 cm^−1^ spectral range. This is more evident in Fig. 4c, d: in these panels, the difference between the second derivative profiles of Ln and Ob samples is shown. An increase in the intensity of specific components of the Ob spectra, compared to Ln spectra, is related to positive peaks of the difference. Larger increases in the intensity for Ob samples are evidenced at 1560, 1740 and 2930 cm^−1^ for RV, and at 1645, 1740 and 2930 cm^−1^ for LV. The negative peak at 1506 cm^−1^ in the difference profile of RV (Fig. 4d) indicates a decrease of intensity for this spectral component passing from Ln to Ob spectra.

#### Mean FTIR spectra and different profiles of ECM films

**Fig. 5:**
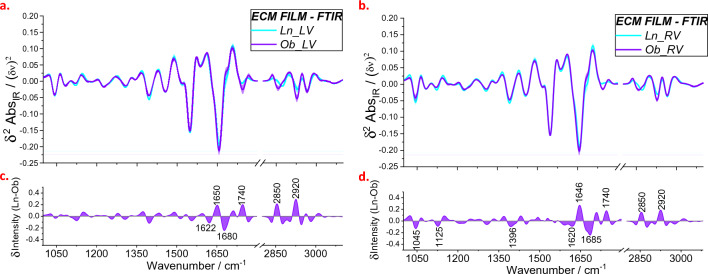
Mean FTIR spectra obtained from tissue prints of the left (**a**) and right (**b**) ventricles of Ln and Ob rats. Difference profiles in panels (**c**) and (**d**) are obtained by subtraction of the violet from the blue curve of panels (**a**) and (**b**), respectively. Data were collected from tissue prints of six lean rats and six obese rats

The ECM was further investigated by imprinting the surface of the fresh section on a CaF_2_ window to achieve the IR spectrum in transmission mode. In Fig. 5, IR measurements of tissue prints are presented. The different spectral components are recognized on the negative intensity of the second derivative spectra shown in Fig. 5a, b; the intensity variation is shown by the difference profiles presented in Fig. 5c, d. The increase or decrease in concentration for a molecular species of Ob compared to Ln spectra is related to the positive or negative peak of difference profiles, respectively. When the whole tissue and ECM assessments are compared, it is remarkable to see that differences are larger for spectra from ECM, and this is predominant for the RV in the regions 2800–2950 cm^−1^ and 1600–1800 cm^−1^. The only exceptions are the peaks at 1506 and 1560 cm^−1^: the intensity variation at these frequencies is larger for the different profiles of the whole tissue rather than ECM film.

## Discussion

ZSF1-Ob rats presented characteristic signs of adverse cardiac remodeling and function. Ob rats exhibited concomitant elevated levels of lipids, triglycerides, glucose, and high blood pressure. Also consistent with the HFpEF phenotype, the Ob rats showed no evidence of compromised systolic function or ventricular dilation, elevated cardiac stiffness and increased interstitial ventricular fibrosis.

In the analysis of cardiac sections, Raman and ATR data showed a different sensitivity toward molecular components of the tissue, though the two techniques both monitor the average chemical composition of the heart. Spectroscopic data suggested that HFpEF is associated with a sensitive change in tissue biochemistry since peaks assigned to cellular and extracellular matrix demonstrated a significant variation of intensity in Ob spectra with respect to samples from Ln rats. Both FTIR and Raman spectra indicated an increase of lipid concentration in the tissues of Ob rats: this was monitored by the Raman bands at 1300 and 1450 cm^−1^, and by the IR signals at 1740, 2850 and 2930 cm^−1^. The enhancement of these bands was almost the same in the spectra from the two ventricles suggesting that this effect is similar for the two chambers. In addition, the increase in lipid concentration was particularly evident in the ECM of both ventricles.

In HFpEF, diastolic dysfunction is often observed due to stiffness of the myocardium and changes in the ECM. The variation of Raman profiles in the regions of amide I (1625–1700 cm^−1^) and amide III (1200–1300 cm^−1^) bands of Ob samples are compatible with a rearrangement of collagen fibres and particularly with an increase of order in the alignment of bundles. This was indicated by the decrease of 1245 cm^−1^ intensity and the corresponding enhancement of 1660 cm^−1^ spectral component [18]. A different result was obtained for the heart tissue of a different animal model of HFpEF, induced by a high salt diet [4]. On tissues from Dalt-sensitive (DSS) rats, an increase of intensity for the amide III band, together with a blue shift of the amide I band was assigned to the increase of collagen concentration in diseased compared to normal animals. In this experiment, rather than an increase in collagen concentration, we observed a structural change in collagen fibres.

Experimental evidence reported in previous studies has shown that the stiffening of cardiac tissue can be related to the altered organization of the collagen network [19]. It was observed that augmented myocardial stiffness is accompanied by structural rather than concentration variations in total myocardial collagen or collagen phenotypes [19]. Abnormal cross-linking between collagen fibers is involved in these structural modifications of the network, which has been referred to as a major determinant of LV diastolic dysfunction in patients with HF. From a pathophysiological point of view, high myocardial collagen cross-linking (CCL) may reduce diastolic reserve and thus, in conditions of fluid overload or exercise, the subsequent acute elevation of filling pressures may lead to reduced diastolic filling and produce HF symptoms [20].

In the Raman spectrum, a possible marker for the presence of CCL is the frequency shift of the amide III band. The increase in cross-linking mechanisms causes collagen fibers to be more tightly connected to each other. This collagen network tightening may limit the rotational freedom of individual fibers, leading to an increase in ordered structures. This effect could explain the narrowing of amide I peak in the IR profile of ECM film, that is evidenced in the difference profiles shown in Fig. 5c, d by the two negative contributions on both high (ca. 1680 cm^−1^) and low (ca. 1620 cm^−1^) frequency sides of amide I maximum at about 1650 cm^−1^.

According to histochemical analysis (Fig. 1), interstitial heart fibrosis of ZSF1 rats is increased in LV and RV of Ob in comparison to Ln (Fig. 1b, c). Perivascular fibrosis appears to be low and similar between Ln and Ob rats (Fig. 1d). Differently, in DSS rats [4] abundant collagen deposition was observed in both perivascular and interstitial areas. Thus, the differences between spectroscopic data obtained for the two models, are coherent with histochemical results.

A pronounced intensity variation between spectra of healthy and diseased samples is found in the 1550–1620 cm^−1^ region of the Raman spectrum. This region is characteristic of vibrations of the aromatic amino acids such as phenylalanine (Phe), tyrosine (Tyr), and tryptophan (Trp), but only Trp shows an intense Raman band at about 1580 cm^−1^ [21]. The significant intensity change at this frequency may therefore be associated with the specific decrease in Trp content in the Ob tissue. This finding provides important information about the inflammatory state of the diseased tissue, which is a favourable condition for the development of HF. In fact, it was observed that the increase in cytokine production induced by the inflammation state is accompanied by a decrease in Trp [22].

The reduction in Trp content may also be related to a different mechanism affecting the stiffness of the tissue cellular component. Trp is an essential component of Titin protein which plays a key role in the contraction and relaxation of cardiomyocytes [23]. A reduction of Trp may result in altered structure and function of Titin leading to increased stiffness [24–26]. We hypothesized that a sign of this effect can be recognized in the band intensity decrease in the 1550–1620 cm^−1^ region of Raman spectra and at 1506 cm^−1^ of the ATR-FTIR spectra, associated with Trp, in the tissue of diseased rats. Our study has detected changes related to cardiac tissue composition in a specific phenotype of HFpEF, in obese ZSF1 rats, aligning with existing knowledge. However, further cellular-level analysis is required. Raman technique can also be used to acquire chemical fingerprints of the main cellular components of the cardiac tissue, although data acquisition and spectra processing require a longer assessment time. Studies of the chemical phenotype of specific cardiac cells, such as cardiomyocytes and cardiac fibroblasts, have the potential for a deeper understanding of the underlying cardiac pathological phenomenon occurring during the evolution of HFpEF.

In ATR, an increase of intensity is observed in the spectral profile of Ob compared to Ln at ~ 1560 cm^−1^, the frequency at which a vibration from glutamate can be observed. The increase in glutamate concentration can be regarded as a marker of HF onset, according to the study by Ariyoshi M. et al. [27]. The mitochondria of cardiac cells contain the protein 9030617O03Rik, whose expression drops significantly in the presence of HF. The reduction of 9030617O03Rik was noted to induce a significant increase in the concentration of glutamate in the heart [27]. The fact that both the augmentation of glutamate and the decrease of Trp concentration relate to the different activity of the cellular constituents of the tissue is confirmed by the absence of relevant intensity variations in the spectra from ECM films.

A difference in the metabolism of Ob vs Ln cells is also indicated by the Raman bands in the region 1000–1100 cm^−1^, which are characteristic of carbohydrates or glycosidic units. Cellular dysfunction underlies the pathogenesis of obesity-associated metabolic disease, including type 2 diabetes. Thus, the presence of high carbohydrate concentration in the Ob cardiac tissue is not surprising. Interestingly, this abnormal carbohydrate concentration is also found in the ECM and this is evidenced by the IR data shown in Fig. 5d.

## Summary

This work demonstrated the potential of vibrational spectroscopic techniques to monitor the biochemical changes present in HFpEF. These spectroscopic tools proved to be very sensitive in detecting changes in the cardiac chemical composition, which may contribute to the alteration in the mechanical properties of this cardiac disease.

Comparison of spectral profiles, particularly Raman spectra, between normal and diseased samples allowed reconstruction of the possible structural and biochemical changes that can in-duce the tissue stiffening. It is reported in the literature that cardiac tissue stiffening is due to variations in both the extracellular and cellular components; in ECM, in particular, a fundamental role is played by collagen. For the specific analysis of ECM, we used tissue printing as a novel analytical tool. The tissue printing method enabled the analysis of ECM as well as the production of replicas of the tissue to perform different analyses. Since these prints can be stored at low temperatures and analyzed later, this approach is especially useful with non-fixed tissue samples.

According to our data, the cardiac tissue of Ob rats does not exhibit an important collagen deposition or type III to type I phenotypic shifts but rather abnormal cross-linking causing the stiffening of ventricles. The stiffening is also evidenced by the decrease of Trp related to the altered Titin structure, and to the inflammatory state which is a favourable condition for HFpEF.

The altered biochemistry of cardiac cells was also recognized in the augmentation of glutamate and glycoproteins monitored in both ventricles but particularly in the right chamber. This asymmetry of altered biochemistry in the two ventricles was observed by IR and Raman analyses of tissues also in a different model of HFpEF [4]. However, in the case of hypertension induced by a high-salt diet, the late stage of HFpEF correlated to the formation of micro-calcifications, which were not observed in the present work. Thus, our study suggests that comorbidities play a fundamental role in organ damage, and a sensitive and selective probe of the tissue biochemistry is fundamental to provide for a specific therapeutic treatment. Overall, these results suggest the presence of specific chemical fingerprints in this model of HFpEF, with increased glutamate and glycoproteins, increased lipids, formation of cross-linked collagen, and decreased Trp content. This differs from the structural change of tyrosine units, the free amino-acid and collagen accumulation, and the formation of cardiac micro-calcifications that have been observed in DSS rats and are perhaps specific to hypertension-induced HF. These findings could aid in better defining the pathological mechanisms underlying different forms of HF. The clinical applicability of these observations will be further increased when the corresponding circulatory fingerprints of these cardiac chemical modifications are determined. Future studies are necessary to identify such early, novel circulatory markers of these spectroscopically detected chemical modifications in cardiac tissue.

## Conclusions and prospective

Vibrational spectroscopy has great potential for clinical applications due to its label-free nature and ability to provide near real-time measurements. However, there are still challenges that need to be addressed, such as low signal and tissue autofluorescence for Raman scattering, and the interference from the strong water signals in FTIR spectra. Techniques derived from the Raman effect or IR absorption (like Optical Photothermal IR [28], Surface Enhanced Raman Scattering [29] or Coherent anti-Stokes Raman scattering [30]), have the potential to enhance the sensitivity and selectivity of tissue analysis and implement their clinical use.

Although there are many challenges, vibrational spectroscopy is a highly versatile, non-invasive, and non-destructive method that has significant potential in the study of cardiovascular disease. Both FTIR and Raman techniques provide valuable information about the specific chemical modifications that occur as the disease progresses and are valid alternatives to histology. Ongoing improvements in instrumentation and data analysis are making these approaches even more effective, and they are expected to be used clinically in the near future as diagnostic tools.

The use of vibrational spectroscopy has the potential to identify specific chemical changes in cardiac tissue, which can help discover new therapeutic targets. Each patient's chemical profile is unique, so the treatment for patients with HFpEF and other diseases may differ based on their chemical fingerprints. Although current diagnostic tools can only classify patients with HFpEF or HFrEF, the identification of unique chemical phenotype in the heart can lead to better patient classification, even if the clinical symptoms are similar across all HFpEF patients.

### Supplementary Information

**Additional file 1: Figure S1.** Fig. S1. Characterization of the ZSF1 model: Body Weight (BW); Tail Length (TL); Systolic Blood Pressure (SBP); Diastolic Blood Pressure (DBP); Ejection Fraction (EF) in Ln and Ob rats. Values are expressed as mean ± SD. * P < 0.05 compared to Ln; *** P < 0.005 compared to Ln; **** P < 0.001 compared to Ln; NS = non-significant. **Figure S2.** Loadings from PCA analysis of Raman data. Data were collected from heart tissues of six Lean and six Obese rats.

## Data Availability

The datasets analysed during the current study are available from the corresponding author upon reasonable request.

## Abbreviations

HFpEF: Heart failure with preserved ejection fraction
FTIR: Fourier-transform infrared
HF: Heart failure
LV: Left ventricular
RV: Right ventricle
ECM: Extracellular matrix
Ob: Obese
Ln: Lean
PVCA/LA: Perivascular collagen area to lumen area
PCA: Principal component analysis
CCL: Collagen cross-linking

## References

[1] Wilson BC, Jermyn M, Leblond F (2018). Challenges and opportunities in clinical translation of biomedical optical spectroscopy and imaging. J Biomed Opt.

[2] Swinson B, Jerjes W, El-Maaytah M (2006). Optical techniques in diagnosis of head and neck malignancy. Oral Oncol.

[3] Zehbe R, Haibel A, Riesemeier H (2010). Going beyond histology. Synchrotron micro-computed tomography as a methodology for biological tissue characterization: from tissue morphology to individual cells. J R Soc Interface.

[4] Tombolesi N, Altara R, da Silva GJJ (2022). Early cardiac-chamber-specific fingerprints in heart failure with preserved ejection fraction detected by FTIR and Raman spectroscopic techniques. Sci Rep.

[5] Lasch Pete, Kneipp J. Biomedical vibrational spectroscopy. 2008; 385

[6] Tiwari S, Reddy VB, Bhargava R, Raman J (2015). Computational chemical imaging for cardiovascular pathology: chemical microscopic imaging accurately determines cardiac transplant rejection. PLoS ONE.

[7] Yamamoto T, Minamikawa T, Harada Y (2018). Label-free evaluation of myocardial infarct in surgically excised ventricular myocardium by Raman spectroscopy. Sci Rep.

[8] John RV, Devasia T, N M (2022). Micro-Raman spectroscopy study of blood samples from myocardial infarction patients. Lasers Med Sci.

[9] Pioppi L, Tombolesi N, Parvan R (2023). FTIR analysis of renal tissue for the assessment of hypertensive organ damage and proANP31&ndash;67 treatment. Int J Mol Sci.

[10] Groenewegen A, Rutten FH, Mosterd A, Hoes AW (2020). Epidemiology of heart failure. Eur J Heart Fail.

[11] Borlaug BA (2020). Evaluation and management of heart failure with preserved ejection fraction. Nat Rev Cardiol.

[12] Gerber Y, Weston SA, Redfield MM (2015). A contemporary appraisal of the heart failure epidemic in Olmsted County, Minnesota, 2000 to 2010. JAMA Intern Med.

[13] Urboniene V, Pucetaite M, Jankevicius F (2014). Identification of kidney tumor tissue by infrared spectroscopy of extracellular matrix. J Biomed Opt.

[14] De Gelder J, De Gussem K, Vandenabeele P, Moens L (2007). Reference database of Raman spectra of biological molecules. J Raman Spectrosc.

[15] Liu C, Boydston-White S, Weisberg A (2016). Vulnerable atherosclerotic plaque detection by resonance Raman spectrosc. J Biomed Opt.

[16] Zohdi V, Whelan DR, Wood BR (2015). Importance of tissue preparation methods in FTIR micro-spectroscopical analysis of biological tissues: ‘traps for new users’. PLoS One.

[17] Nguyen ITN, Brandt MM, van de Wouw J (2020). Both male and female obese ZSF1 rats develop cardiac dysfunction in obesity-induced heart failure with preserved ejection fraction. PLoS One.

[18] Bonifacio A, Sergo V (2010). Effects of sample orientation in Raman microspectroscopy of collagen fibers and their impact on the interpretation of the amide III band. Vib Spectrosc.

[19] Norton GR, Tsotetsi J, Trifunovic B (1997). Myocardial stiffness is attributed to alterations in cross-linked collagen rather than total collagen or phenotypes in spontaneously hypertensive rats. Circulation.

[20] López B, Ravassa S, González A (2016). Myocardial collagen cross-linking is associated with heart failure hospitalization in patients with hypertensive heart failure. J Am Coll Cardiol.

[21] Chi Z, Chen XG, Holtz JSW, Asher SA (1998). UV resonance Raman-selective amide vibrational enhancement: quantitative methodology for determining protein secondary structure. Biochemistry.

[22] Mangge H, Stelzer I, Reininghaus EZ (2014). Disturbed tryptophan metabolism in cardiovascular disease. Curr Med Chem.

[23] Vattepu R, Klausmeyer RA, Ayella A (2020). Conserved tryptophan mutation disrupts structure and function of immunoglobulin domain revealing unusual tyrosine fluorescence. Protein Sci.

[24] Somkuti J, Mártonfalvi Z, Kellermayer MSZ, Smeller L (2013). Different pressure–temperature behavior of the structured and unstructured regions of titin. Biochimica Biophysica Acta.

[25] Hochman-Mendez C, Curty E, Taylor DA (2020). Change the laminin, change the cardiomyocyte: improve untreatable heart failure. Int J Mol Sci.

[26] Borbély A, Falcao-Pires I, Van Heerebeek L (2009). Hypophosphorylation of the stiff N2B Titin isoform raises cardiomyocyte resting tension in failing human myocardium. Circ Res.

[27] Ariyoshi M, Katane M, Hamase K (2017). D-Glutamate is metabolized in the heart mitochondria. Sci Rep.

[28] Klementieva O, Sandt C, Martinsson I, Kansiz M, Gouras GK, Borondics F (2020). Super-resolution infrared imaging of polymorphic amyloid aggregates directly in neurons. Adv Sci.

[29] Han XX, Rodriguez RS, Haynes CL, Ozaki Y, Zhao B (2022). Surface-enhanced Raman spectroscopy. Nat Rev Methods Primer.

[30] Vernuccio F, Vanna R, Ceconello C, Bresci A, Manetti F, Sorrentino S (2023). Full-Spectrum CARS microscopy of cells and tissues with ultrashort white-light continuum pulses. J Phys Chem B.

