# Supplementary material for: Vibrational spectroscopy identifies myocardial chemical modifications in heart failure with preserved ejection fraction
Source: J Transl Med. 2023 Sep 11;21:617. doi: 10.1186/s12967-023-04465-0 (PMC10496315; doi:10.1186/s12967-023-04465-0)
Supplement: Supplementary file 1 — Additional file 1: Figure S1. Fig. S1. Characterization of the ZSF1 model: Body Weight (BW); Tail Length (TL); Systolic Blood Pressure (SBP); Diastolic Blood Pressure (DBP); Ejection Fraction (EF) in Ln and Ob rats. Values are expressed as mean ± SD. * P < 0.05 compared to Ln; *** P < 0.005 compared to Ln; **** P < 0.001 compared to Ln; NS = non-significant. Figure S2. Loadings from PCA analysis of Raman data. Data were collected from heart tissues of six Lean and six Obese rats. [file 12967_2023_4465_MOESM1_ESM.docx]

**Additional Material**

**A**

**B**

**C**

**D**

**E**

**G**

**F**

**H**

**I**

**Fig. S1. Characterization of the ZSF1 model:** Body Weight (BW); Tail Length (TL); Systolic Blood Pressure (SBP); Diastolic Blood Pressure (DBP); Ejection Fraction (EF) in Ln and Ob rats. Values are expressed as mean ± SD. * P < 0.05 compared to Ln; *** P < 0.005 compared to Ln; **** P < 0.001 compared to Ln; NS = non-significant.

**
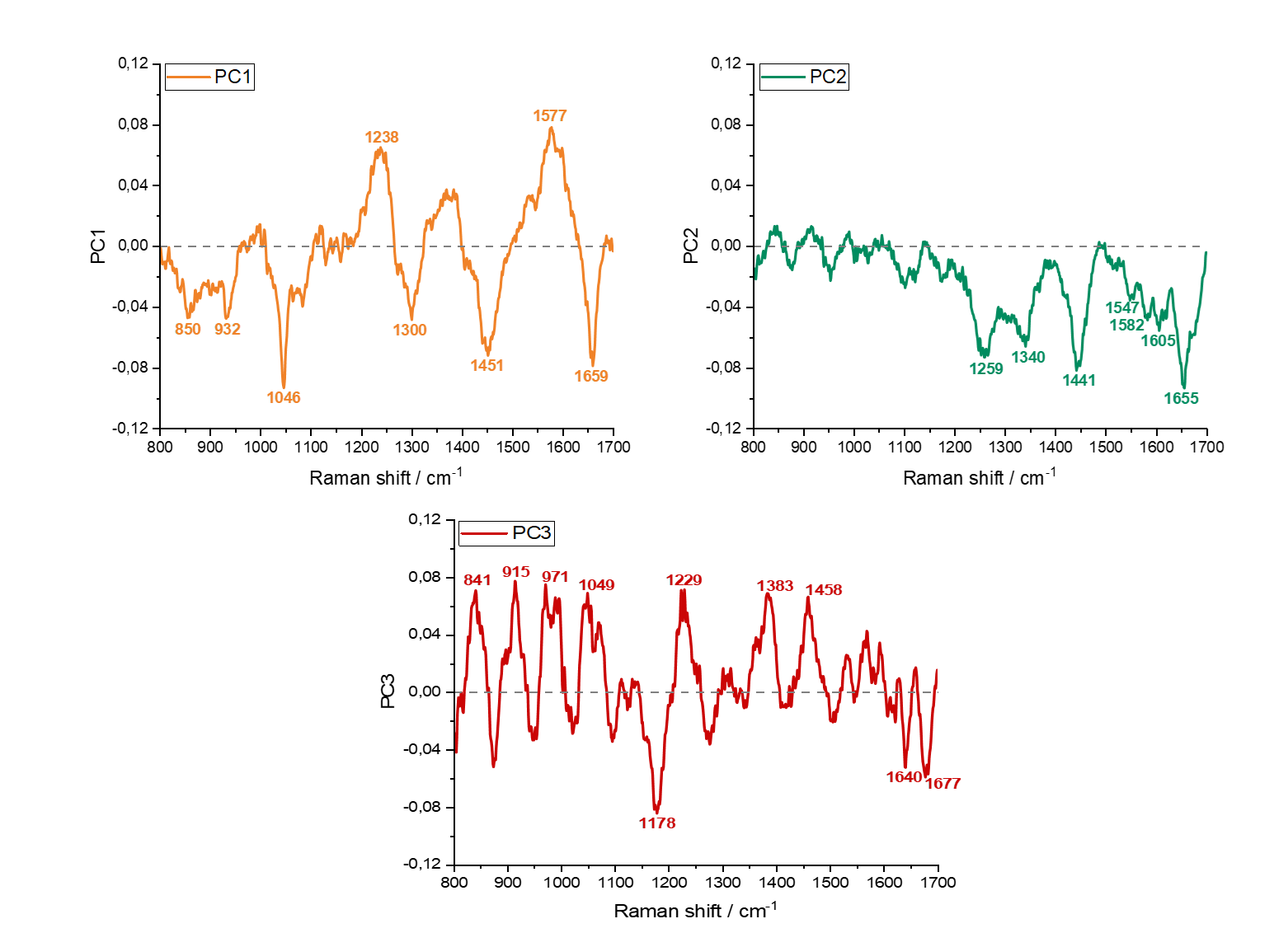
**

**Fig. S2. Loadings from PCA analysis of Raman data.** Data were collected from heart tissues of six Lean and six Obese rats.

Principal Component Analysis is a commonly used multivariate approach able to reduce the dimensionality of large datasets. It can be applied to spectroscopic data to identify the most variable spectral features of a sample (or sample’s area) based on differences in biochemical content. In this approach, loadings represent spectral variations which differentiate the groupings according to the intensity measured at each wavenumber.

The two groups of cardiac tissues from Ln and Ob rats are well discriminated using this method, and the basis for discrimination is derived from analysis of the loadings corresponding to each PC. The minima and maxima of each PC are analyzed in order to recognize the spectral components (bands) of the pristine spectrum; differently from spectra, the bands of loadings can be either positive or negative. The molecular species constituting the tissue are assigned to these components. A single PC does not necessarily represent a single molecular species, since different types of spectra can be distinguished by the variation of several bands with respect to others. These means that different types of tissue can be distinguished because more than one molecular species vary with respect to others.

The PC1 loading reported above shows the signals characteristic of tryptophane (1577 cm^-1^), proteins (930, 1238, 1300 and 1659 cm^-1^), lipids (1451 cm^-1^) and glycoproteins (1046 cm^-1^). The opposite sign of 1577 and 1238 cm^-1^ features with respect to others suggests that Trp (1577 cm^-1^) and less ordered structures of collagen (1238 cm^-1^) show an opposite variation with respect to the other molecular species (highly ordered collagen fibers, lipids and glycoproteins). In Ob-LV samples, the two features decrease, and the others increase in comparison to Ln-LV samples. The clustering of RV tissues is also depending on the intensity change at 1178 cm^-1^ (intense negative feature of PC3) which is assigned to proteins.
